# Supplementary material for: Atom Identifiers Generated by a Neighborhood-Specific Graph Coloring Method Enable Compound Harmonization across Metabolic Databases
Source: Metabolites. 2020 Sep 11;10(9):368. doi: 10.3390/metabo10090368 (PMC7570338; doi:10.3390/metabo10090368)
Supplement: Supplementary file 1 [file metabolites-10-00368-s001.zip › supplementary_materials_v7.2.docx]

**Supporting Information for**

Atom Identifiers Generated by a Neighborhood-Specific Graph Coloring Method Enable Compound Harmonization Across Metabolic Databases

Huan Jin ^1^, Joshua M. Mitchell ^2,3,4^ and Hunter N.B. Moseley ^2, 3, 4, 5,^ *

^1^ Department of Toxicology and Cancer Biology, University of Kentucky, Lexington, KY 40536, USA; huan.jin@uky.edu

^2^ Department of Molecular & Cellular Biochemistry, University of Kentucky, Lexington, KY 40536, USA

^3^ Markey Cancer Center, University of Kentucky, Lexington, KY 40536, USA

^4^ Resource Center for Stable Isotope Resolved Metabolomics, University of Kentucky, Lexington, KY 40536, USA

^5^ Institute for Biomedical Informatics, University of Kentucky, Lexington, KY 40536, USA

***** Correspondence: hunter.moseley@uky.edu; Tel.: 859-218-2964

"(1)(C(C(C,1)(C,1))(C(C,1)(C,4)(C,4)C(C,1)(O,1)(O,2))(C(C,1)(C,1)C(C,1)(C,1)C(C,4)(C,4)C(C,4)(C,4)(C,4)O(C,1)O(C,2))(C(C,1)(C,4)(C,4)C(C,1)(C,4)(C,4)C(C,1)(O,1)(O,2)C(C,1)(O,1)(O,2)C(C,4)(C,4)C(C,4)(C,4)C(C,4)(C,4)(C,4))(C(C,4)(C,4)C(C,4)(C,4)C(C,4)(C,4)C(C,4)(C,4)C(C,4)(C,4)C(C,4)(C,4)(C,4)C(C,4)(C,4)(C,4)))(1)(C(C(C,1)(C,4)(C,4))(C(C,1)(C,1)C(C,4)(C,4)C(C,4)(C,4)(C,4))(C(C,1)(C,4)(C,4)C(C,1)(C,4)(C,4)C(C,1)(C,4)(C,4)C(C,1)(O,1)(O,2)C(C,4)(C,4)C(C,4)(C,4)C(C,4)(C,4)(C,4))(C(C,1)(C,1)C(C,4)(C,4)C(C,4)(C,4)C(C,4)(C,4)C(C,4)(C,4)C(C,4)(C,4)C(C,4)(C,4)(C,4)C(C,4)(C,4)(C,4)O(C,1)O(C,2))(C(C,1)(O,1)(O,2)C(C,1)(O,1)(O,2)C(C,4)(C,4)C(C,4)(C,4)C(C,4)(C,4)C(C,4)(C,4)C(C,4)(C,4)(C,4)C(C,4)(C,4)(C,4)))(1)(C(C(C,1)(O,1)(O,2))(C(C,1)(C,1)O(C,1)O(C,2))(C(C,1)(C,4)(C,4)C(C,1)(O,1)(O,2)C(C,1)(O,1)(O,2)C(C,1)(O,1)(O,2))(C(C,1)(C,1)C(C,4)(C,4)C(C,4)(C,4)(C,4))(C(C,1)(C,4)(C,4)C(C,1)(C,4)(C,4)C(C,4)(C,4)C(C,4)(C,4)C(C,4)(C,4)(C,4)))(1)(C(C(C,4)(C,4)(C,4))(C(C,1)(C,4)(C,4)C(C,4)(C,4)C(C,4)(C,4)(C,4))(C(C,1)(C,1)C(C,4)(C,4)C(C,4)(C,4)C(C,4)(C,4)C(C,4)(C,4)C(C,4)(C,4)(C,4)C(C,4)(C,4)(C,4)C(C,4)(C,4)(C,4))(C(C,1)(C,4)(C,4)C(C,1)(C,4)(C,4)C(C,1)(O,1)(O,2)C(C,4)(C,4)C(C,4)(C,4)C(C,4)(C,4)C(C,4)(C,4)C(C,4)(C,4)C(C,4)(C,4)(C,4)C(C,4)(C,4)(C,4))(C(C,1)(C,1)C(C,4)(C,4)C(C,4)(C,4)C(C,4)(C,4)C(C,4)(C,4)O(C,1)O(C,2)))(1)(C(C(C,4)(C,4)(C,4))(C(C,4)(C,4)C(C,4)(C,4)C(C,4)(C,4)(C,4))(C(C,1)(C,4)(C,4)C(C,4)(C,4)C(C,4)(C,4)C(C,4)(C,4)C(C,4)(C,4)(C,4)C(C,4)(C,4)(C,4)C(C,4)(C,4)(C,4))(C(C,1)(C,1)C(C,4)(C,4)C(C,4)(C,4)C(C,4)(C,4)C(C,4)(C,4)C(C,4)(C,4)C(C,4)(C,4)C(C,4)(C,4)(C,4)C(C,4)(C,4)(C,4))(C(C,1)(C,4)(C,4)C(C,1)(C,4)(C,4)C(C,1)(O,1)(O,2)C(C,4)(C,4)C(C,4)(C,4)C(C,4)(C,4)))(1)(C(C(C,4)(C,4))(C(C,1)(C,4)(C,4)C(C,4)(C,4))(C(C,1)(C,1)C(C,4)(C,4)C(C,4)(C,4)C(C,4)(C,4)C(C,4)(C,4)(C,4))(C(C,1)(C,4)(C,4)C(C,1)(C,4)(C,4)C(C,1)(O,1)(O,2)C(C,4)(C,4)C(C,4)(C,4)C(C,4)(C,4)(C,4)C(C,4)(C,4)(C,4))(C(C,1)(C,1)C(C,4)(C,4)C(C,4)(C,4)C(C,4)(C,4)C(C,4)(C,4)(C,4)C(C,4)(C,4)(C,4)O(C,1)O(C,2)))(1)(C(C(C,4)(C,4))(C(C,4)(C,4)C(C,4)(C,4)(C,4))(C(C,1)(C,4)(C,4)C(C,4)(C,4)C(C,4)(C,4)C(C,4)(C,4)C(C,4)(C,4)(C,4))(C(C,1)(C,1)C(C,4)(C,4)C(C,4)(C,4)C(C,4)(C,4)C(C,4)(C,4)C(C,4)(C,4)C(C,4)(C,4)(C,4)C(C,4)(C,4)(C,4))(C(C,1)(C,4)(C,4)C(C,1)(C,4)(C,4)C(C,1)(O,1)(O,2)C(C,4)(C,4)C(C,4)(C,4)C(C,4)(C,4)C(C,4)(C,4)(C,4)C(C,4)(C,4)(C,4)))(1)(C(C(C,4)(C,4))(C(C,4)(C,4)C(C,4)(C,4)(C,4))(C(C,4)(C,4)C(C,4)(C,4)C(C,4)(C,4)C(C,4)(C,4)C(C,4)(C,4)(C,4))(C(C,1)(C,4)(C,4)C(C,1)(C,4)(C,4)C(C,4)(C,4)C(C,4)(C,4)C(C,4)(C,4)C(C,4)(C,4)(C,4)C(C,4)(C,4)(C,4))(C(C,1)(C,1)C(C,4)(C,4)C(C,4)(C,4)C(C,4)(C,4)C(C,4)(C,4)C(C,4)(C,4)(C,4)C(C,4)(C,4)(C,4)))(1)(C(C(C,4)(C,4))(C(C,4)(C,4)C(C,4)(C,4)(C,4))(C(C,4)(C,4)C(C,4)(C,4)C(C,4)(C,4)C(C,4)(C,4)C(C,4)(C,4)(C,4))(C(C,1)(C,4)(C,4)C(C,4)(C,4)C(C,4)(C,4)C(C,4)(C,4)C(C,4)(C,4)C(C,4)(C,4)(C,4)C(C,4)(C,4)(C,4))(C(C,1)(C,1)C(C,4)(C,4)C(C,4)(C,4)C(C,4)(C,4)C(C,4)(C,4)C(C,4)(C,4)(C,4)C(C,4)(C,4)(C,4)))(1)(C(C(C,4)(C,4))(C(C,4)(C,4)C(C,4)(C,4))(C(C,1)(C,4)(C,4)C(C,4)(C,4)C(C,4)(C,4)C(C,4)(C,4)(C,4))(C(C,1)(C,1)C(C,4)(C,4)C(C,4)(C,4)C(C,4)(C,4)C(C,4)(C,4)(C,4)C(C,4)(C,4)(C,4))(C(C,1)(C,4)(C,4)C(C,1)(C,4)(C,4)C(C,1)(O,1)(O,2)C(C,4)(C,4)C(C,4)(C,4)C(C,4)(C,4)(C,4)C(C,4)(C,4)(C,4)))(1)(C(C(C,4)(C,4))(C(C,4)(C,4)C(C,4)(C,4))(C(C,4)(C,4)C(C,4)(C,4)C(C,4)(C,4)C(C,4)(C,4)(C,4))(C(C,1)(C,4)(C,4)C(C,4)(C,4)C(C,4)(C,4)C(C,4)(C,4)(C,4)C(C,4)(C,4)(C,4))(C(C,1)(C,1)C(C,4)(C,4)C(C,4)(C,4)C(C,4)(C,4)C(C,4)(C,4)(C,4)C(C,4)(C,4)(C,4)))(1)(C(C(C,4)(C,4))(C(C,4)(C,4)C(C,4)(C,4))(C(C,4)(C,4)C(C,4)(C,4)C(C,4)(C,4)C(C,4)(C,4)(C,4))(C(C,4)(C,4)C(C,4)(C,4)C(C,4)(C,4)C(C,4)(C,4)(C,4)C(C,4)(C,4)(C,4))(C(C,1)(C,4)(C,4)C(C,4)(C,4)C(C,4)(C,4)C(C,4)(C,4)(C,4)C(C,4)(C,4)(C,4)))(1)(O(O(C,1))(C(C,1)(O,1)(O,2))(C(C,1)(C,1)O(C,1)O(C,2))(C(C,1)(C,4)(C,4)C(C,1)(O,1)(O,2)C(C,1)(O,1)(O,2))(C(C,1)(C,1)C(C,4)(C,4)C(C,4)(C,4)(C,4)))(1)(O(O(C,2))(C(C,1)(O,1)(O,2))(C(C,1)(C,1)O(C,1)O(C,2))(C(C,1)(C,4)(C,4)C(C,1)(O,1)(O,2)C(C,1)(O,1)(O,2))(C(C,1)(C,1)C(C,4)(C,4)C(C,4)(C,4)(C,4)))"

A.

"(1)(C000(C000(C000,10)(C000,10))(C000(C000,10)(C000,40)(C000,40)C000(C000,10)(O000,10)(O000,20))(C000(C000,10)(C000,10)C000(C000,10)(C000,10)C000(C000,40)(C000,40)C000(C000,40)(C000,40)(C000,40)O000(C000,10)O000(C000,20))(C000(C000,10)(C000,40)(C000,40)C000(C000,10)(C000,40)(C000,40)C000(C000,10)(O000,10)(O000,20)C000(C000,10)(O000,10)(O000,20)C000(C000,40)(C000,40)C000(C000,40)(C000,40)C000(C000,40)(C000,40)(C000,40))(C000(C000,40)(C000,40)C000(C000,40)(C000,40)C000(C000,40)(C000,40)C000(C000,40)(C000,40)C000(C000,40)(C000,40)C000(C000,40)(C000,40)(C000,40)C000(C000,40)(C000,40)(C000,40)))(1)(C000(C000(C000,10)(C000,40)(C000,40))(C000(C000,10)(C000,10)C000(C000,40)(C000,40)C000(C000,40)(C000,40)(C000,40))(C000(C000,10)(C000,40)(C000,40)C000(C000,10)(C000,40)(C000,40)C000(C000,10)(C000,40)(C000,40)C000(C000,10)(O000,10)(O000,20)C000(C000,40)(C000,40)C000(C000,40)(C000,40)C000(C000,40)(C000,40)(C000,40))(C000(C000,10)(C000,10)C000(C000,40)(C000,40)C000(C000,40)(C000,40)C000(C000,40)(C000,40)C000(C000,40)(C000,40)C000(C000,40)(C000,40)C000(C000,40)(C000,40)(C000,40)C000(C000,40)(C000,40)(C000,40)O000(C000,10)O000(C000,20))(C000(C000,10)(O000,10)(O000,20)C000(C000,10)(O000,10)(O000,20)C000(C000,40)(C000,40)C000(C000,40)(C000,40)C000(C000,40)(C000,40)C000(C000,40)(C000,40)C000(C000,40)(C000,40)(C000,40)C000(C000,40)(C000,40)(C000,40)))(1)(C000(C000(C000,10)(O000,10)(O000,20))(C000(C000,10)(C000,10)O000(C000,10)O000(C000,20))(C000(C000,10)(C000,40)(C000,40)C000(C000,10)(O000,10)(O000,20)C000(C000,10)(O000,10)(O000,20)C000(C000,10)(O000,10)(O000,20))(C000(C000,10)(C000,10)C000(C000,40)(C000,40)C000(C000,40)(C000,40)(C000,40))(C000(C000,10)(C000,40)(C000,40)C000(C000,10)(C000,40)(C000,40)C000(C000,40)(C000,40)C000(C000,40)(C000,40)C000(C000,40)(C000,40)(C000,40)))(1)(C000(C000(C000,40)(C000,40)(C000,40))(C000(C000,10)(C000,40)(C000,40)C000(C000,40)(C000,40)C000(C000,40)(C000,40)(C000,40))(C000(C000,10)(C000,10)C000(C000,40)(C000,40)C000(C000,40)(C000,40)C000(C000,40)(C000,40)C000(C000,40)(C000,40)C000(C000,40)(C000,40)(C000,40)C000(C000,40)(C000,40)(C000,40)C000(C000,40)(C000,40)(C000,40))(C000(C000,10)(C000,40)(C000,40)C000(C000,10)(C000,40)(C000,40)C000(C000,10)(O000,10)(O000,20)C000(C000,40)(C000,40)C000(C000,40)(C000,40)C000(C000,40)(C000,40)C000(C000,40)(C000,40)C000(C000,40)(C000,40)C000(C000,40)(C000,40)(C000,40)C000(C000,40)(C000,40)(C000,40))(C000(C000,10)(C000,10)C000(C000,40)(C000,40)C000(C000,40)(C000,40)C000(C000,40)(C000,40)C000(C000,40)(C000,40)O000(C000,10)O000(C000,20)))(1)(C000(C000(C000,40)(C000,40)(C000,40))(C000(C000,40)(C000,40)C000(C000,40)(C000,40)C000(C000,40)(C000,40)(C000,40))(C000(C000,10)(C000,40)(C000,40)C000(C000,40)(C000,40)C000(C000,40)(C000,40)C000(C000,40)(C000,40)C000(C000,40)(C000,40)(C000,40)C000(C000,40)(C000,40)(C000,40)C000(C000,40)(C000,40)(C000,40))(C000(C000,10)(C000,10)C000(C000,40)(C000,40)C000(C000,40)(C000,40)C000(C000,40)(C000,40)C000(C000,40)(C000,40)C000(C000,40)(C000,40)C000(C000,40)(C000,40)C000(C000,40)(C000,40)(C000,40)C000(C000,40)(C000,40)(C000,40))(C000(C000,10)(C000,40)(C000,40)C000(C000,10)(C000,40)(C000,40)C000(C000,10)(O000,10)(O000,20)C000(C000,40)(C000,40)C000(C000,40)(C000,40)C000(C000,40)(C000,40)))(1)(C000(C000(C000,40)(C000,40))(C000(C000,10)(C000,40)(C000,40)C000(C000,40)(C000,40))(C000(C000,10)(C000,10)C000(C000,40)(C000,40)C000(C000,40)(C000,40)C000(C000,40)(C000,40)C000(C000,40)(C000,40)(C000,40))(C000(C000,10)(C000,40)(C000,40)C000(C000,10)(C000,40)(C000,40)C000(C000,10)(O000,10)(O000,20)C000(C000,40)(C000,40)C000(C000,40)(C000,40)C000(C000,40)(C000,40)(C000,40)C000(C000,40)(C000,40)(C000,40))(C000(C000,10)(C000,10)C000(C000,40)(C000,40)C000(C000,40)(C000,40)C000(C000,40)(C000,40)C000(C000,40)(C000,40)(C000,40)C000(C000,40)(C000,40)(C000,40)O000(C000,10)O000(C000,20)))(1)(C000(C000(C000,40)(C000,40))(C000(C000,40)(C000,40)C000(C000,40)(C000,40)(C000,40))(C000(C000,10)(C000,40)(C000,40)C000(C000,40)(C000,40)C000(C000,40)(C000,40)C000(C000,40)(C000,40)C000(C000,40)(C000,40)(C000,40))(C000(C000,10)(C000,10)C000(C000,40)(C000,40)C000(C000,40)(C000,40)C000(C000,40)(C000,40)C000(C000,40)(C000,40)C000(C000,40)(C000,40)C000(C000,40)(C000,40)(C000,40)C000(C000,40)(C000,40)(C000,40))(C000(C000,10)(C000,40)(C000,40)C000(C000,10)(C000,40)(C000,40)C000(C000,10)(O000,10)(O000,20)C000(C000,40)(C000,40)C000(C000,40)(C000,40)C000(C000,40)(C000,40)C000(C000,40)(C000,40)(C000,40)C000(C000,40)(C000,40)(C000,40)))(1)(C000(C000(C000,40)(C000,40))(C000(C000,40)(C000,40)C000(C000,40)(C000,40)(C000,40))(C000(C000,40)(C000,40)C000(C000,40)(C000,40)C000(C000,40)(C000,40)C000(C000,40)(C000,40)C000(C000,40)(C000,40)(C000,40))(C000(C000,10)(C000,40)(C000,40)C000(C000,10)(C000,40)(C000,40)C000(C000,40)(C000,40)C000(C000,40)(C000,40)C000(C000,40)(C000,40)C000(C000,40)(C000,40)(C000,40)C000(C000,40)(C000,40)(C000,40))(C000(C000,10)(C000,10)C000(C000,40)(C000,40)C000(C000,40)(C000,40)C000(C000,40)(C000,40)C000(C000,40)(C000,40)C000(C000,40)(C000,40)(C000,40)C000(C000,40)(C000,40)(C000,40)))(1)(C000(C000(C000,40)(C000,40))(C000(C000,40)(C000,40)C000(C000,40)(C000,40)(C000,40))(C000(C000,40)(C000,40)C000(C000,40)(C000,40)C000(C000,40)(C000,40)C000(C000,40)(C000,40)C000(C000,40)(C000,40)(C000,40))(C000(C000,10)(C000,40)(C000,40)C000(C000,40)(C000,40)C000(C000,40)(C000,40)C000(C000,40)(C000,40)C000(C000,40)(C000,40)C000(C000,40)(C000,40)(C000,40)C000(C000,40)(C000,40)(C000,40))(C000(C000,10)(C000,10)C000(C000,40)(C000,40)C000(C000,40)(C000,40)C000(C000,40)(C000,40)C000(C000,40)(C000,40)C000(C000,40)(C000,40)(C000,40)C000(C000,40)(C000,40)(C000,40)))(1)(C000(C000(C000,40)(C000,40))(C000(C000,40)(C000,40)C000(C000,40)(C000,40))(C000(C000,10)(C000,40)(C000,40)C000(C000,40)(C000,40)C000(C000,40)(C000,40)C000(C000,40)(C000,40)(C000,40))(C000(C000,10)(C000,10)C000(C000,40)(C000,40)C000(C000,40)(C000,40)C000(C000,40)(C000,40)C000(C000,40)(C000,40)(C000,40)C000(C000,40)(C000,40)(C000,40))(C000(C000,10)(C000,40)(C000,40)C000(C000,10)(C000,40)(C000,40)C000(C000,10)(O000,10)(O000,20)C000(C000,40)(C000,40)C000(C000,40)(C000,40)C000(C000,40)(C000,40)(C000,40)C000(C000,40)(C000,40)(C000,40)))(1)(C000(C000(C000,40)(C000,40))(C000(C000,40)(C000,40)C000(C000,40)(C000,40))(C000(C000,40)(C000,40)C000(C000,40)(C000,40)C000(C000,40)(C000,40)C000(C000,40)(C000,40)(C000,40))(C000(C000,10)(C000,40)(C000,40)C000(C000,40)(C000,40)C000(C000,40)(C000,40)C000(C000,40)(C000,40)(C000,40)C000(C000,40)(C000,40)(C000,40))(C000(C000,10)(C000,10)C000(C000,40)(C000,40)C000(C000,40)(C000,40)C000(C000,40)(C000,40)C000(C000,40)(C000,40)(C000,40)C000(C000,40)(C000,40)(C000,40)))(1)(C000(C000(C000,40)(C000,40))(C000(C000,40)(C000,40)C000(C000,40)(C000,40))(C000(C000,40)(C000,40)C000(C000,40)(C000,40)C000(C000,40)(C000,40)C000(C000,40)(C000,40)(C000,40))(C000(C000,40)(C000,40)C000(C000,40)(C000,40)C000(C000,40)(C000,40)C000(C000,40)(C000,40)(C000,40)C000(C000,40)(C000,40)(C000,40))(C000(C000,10)(C000,40)(C000,40)C000(C000,40)(C000,40)C000(C000,40)(C000,40)C000(C000,40)(C000,40)(C000,40)C000(C000,40)(C000,40)(C000,40)))(1)(O000(O000(C000,10))(C000(C000,10)(O000,10)(O000,20))(C000(C000,10)(C000,10)O000(C000,10)O000(C000,20))(C000(C000,10)(C000,40)(C000,40)C000(C000,10)(O000,10)(O000,20)C000(C000,10)(O000,10)(O000,20))(C000(C000,10)(C000,10)C000(C000,40)(C000,40)C000(C000,40)(C000,40)(C000,40)))(1)(O000(O000(C000,20))(C000(C000,10)(O000,10)(O000,20))(C000(C000,10)(C000,10)O000(C000,10)O000(C000,20))(C000(C000,10)(C000,40)(C000,40)C000(C000,10)(O000,10)(O000,20)C000(C000,10)(O000,10)(O000,20))(C000(C000,10)(C000,10)C000(C000,40)(C000,40)C000(C000,40)(C000,40)(C000,40)))"

B.

"(1)(C000(C000(C000,10)(C000,10))(C000(C000,10)(C000,40)(C000,40)C000(C000,10)(O0-10,10)(O000,20))(C000(C000,10)(C000,10)C000(C000,10)(C000,10)C000(C000,40)(C000,40)C000(C000,40)(C000,40)(C000,40)O0-10(C000,10)O000(C000,20))(C000(C000,10)(C000,40)(C000,40)C000(C000,10)(C000,40)(C000,40)C000(C000,10)(O0-10,10)(O000,20)C000(C000,10)(O0-10,10)(O000,20)C000(C000,40)(C000,40)C000(C000,40)(C000,40)C000(C000,40)(C000,40)(C000,40))(C000(C000,40)(C000,40)C000(C000,40)(C000,40)C000(C000,40)(C000,40)C000(C000,40)(C000,40)C000(C000,40)(C000,40)C000(C000,40)(C000,40)(C000,40)C000(C000,40)(C000,40)(C000,40)))(1)(C000(C000(C000,10)(C000,40)(C000,40))(C000(C000,10)(C000,10)C000(C000,40)(C000,40)C000(C000,40)(C000,40)(C000,40))(C000(C000,10)(C000,40)(C000,40)C000(C000,10)(C000,40)(C000,40)C000(C000,10)(C000,40)(C000,40)C000(C000,10)(O0-10,10)(O000,20)C000(C000,40)(C000,40)C000(C000,40)(C000,40)C000(C000,40)(C000,40)(C000,40))(C000(C000,10)(C000,10)C000(C000,40)(C000,40)C000(C000,40)(C000,40)C000(C000,40)(C000,40)C000(C000,40)(C000,40)C000(C000,40)(C000,40)C000(C000,40)(C000,40)(C000,40)C000(C000,40)(C000,40)(C000,40)O0-10(C000,10)O000(C000,20))(C000(C000,10)(O0-10,10)(O000,20)C000(C000,10)(O0-10,10)(O000,20)C000(C000,40)(C000,40)C000(C000,40)(C000,40)C000(C000,40)(C000,40)C000(C000,40)(C000,40)C000(C000,40)(C000,40)(C000,40)C000(C000,40)(C000,40)(C000,40)))(1)(C000(C000(C000,10)(O0-10,10)(O000,20))(C000(C000,10)(C000,10)O0-10(C000,10)O000(C000,20))(C000(C000,10)(C000,40)(C000,40)C000(C000,10)(O0-10,10)(O000,20)C000(C000,10)(O0-10,10)(O000,20)C000(C000,10)(O0-10,10)(O000,20))(C000(C000,10)(C000,10)C000(C000,40)(C000,40)C000(C000,40)(C000,40)(C000,40))(C000(C000,10)(C000,40)(C000,40)C000(C000,10)(C000,40)(C000,40)C000(C000,40)(C000,40)C000(C000,40)(C000,40)C000(C000,40)(C000,40)(C000,40)))(1)(C000(C000(C000,40)(C000,40)(C000,40))(C000(C000,10)(C000,40)(C000,40)C000(C000,40)(C000,40)C000(C000,40)(C000,40)(C000,40))(C000(C000,10)(C000,10)C000(C000,40)(C000,40)C000(C000,40)(C000,40)C000(C000,40)(C000,40)C000(C000,40)(C000,40)C000(C000,40)(C000,40)(C000,40)C000(C000,40)(C000,40)(C000,40)C000(C000,40)(C000,40)(C000,40))(C000(C000,10)(C000,40)(C000,40)C000(C000,10)(C000,40)(C000,40)C000(C000,10)(O0-10,10)(O000,20)C000(C000,40)(C000,40)C000(C000,40)(C000,40)C000(C000,40)(C000,40)C000(C000,40)(C000,40)C000(C000,40)(C000,40)C000(C000,40)(C000,40)(C000,40)C000(C000,40)(C000,40)(C000,40))(C000(C000,10)(C000,10)C000(C000,40)(C000,40)C000(C000,40)(C000,40)C000(C000,40)(C000,40)C000(C000,40)(C000,40)O0-10(C000,10)O000(C000,20)))(1)(C000(C000(C000,40)(C000,40)(C000,40))(C000(C000,40)(C000,40)C000(C000,40)(C000,40)C000(C000,40)(C000,40)(C000,40))(C000(C000,10)(C000,40)(C000,40)C000(C000,40)(C000,40)C000(C000,40)(C000,40)C000(C000,40)(C000,40)C000(C000,40)(C000,40)(C000,40)C000(C000,40)(C000,40)(C000,40)C000(C000,40)(C000,40)(C000,40))(C000(C000,10)(C000,10)C000(C000,40)(C000,40)C000(C000,40)(C000,40)C000(C000,40)(C000,40)C000(C000,40)(C000,40)C000(C000,40)(C000,40)C000(C000,40)(C000,40)C000(C000,40)(C000,40)(C000,40)C000(C000,40)(C000,40)(C000,40))(C000(C000,10)(C000,40)(C000,40)C000(C000,10)(C000,40)(C000,40)C000(C000,10)(O0-10,10)(O000,20)C000(C000,40)(C000,40)C000(C000,40)(C000,40)C000(C000,40)(C000,40)))(1)(C000(C000(C000,40)(C000,40))(C000(C000,10)(C000,40)(C000,40)C000(C000,40)(C000,40))(C000(C000,10)(C000,10)C000(C000,40)(C000,40)C000(C000,40)(C000,40)C000(C000,40)(C000,40)C000(C000,40)(C000,40)(C000,40))(C000(C000,10)(C000,40)(C000,40)C000(C000,10)(C000,40)(C000,40)C000(C000,10)(O0-10,10)(O000,20)C000(C000,40)(C000,40)C000(C000,40)(C000,40)C000(C000,40)(C000,40)(C000,40)C000(C000,40)(C000,40)(C000,40))(C000(C000,10)(C000,10)C000(C000,40)(C000,40)C000(C000,40)(C000,40)C000(C000,40)(C000,40)C000(C000,40)(C000,40)(C000,40)C000(C000,40)(C000,40)(C000,40)O0-10(C000,10)O000(C000,20)))(1)(C000(C000(C000,40)(C000,40))(C000(C000,40)(C000,40)C000(C000,40)(C000,40)(C000,40))(C000(C000,10)(C000,40)(C000,40)C000(C000,40)(C000,40)C000(C000,40)(C000,40)C000(C000,40)(C000,40)C000(C000,40)(C000,40)(C000,40))(C000(C000,10)(C000,10)C000(C000,40)(C000,40)C000(C000,40)(C000,40)C000(C000,40)(C000,40)C000(C000,40)(C000,40)C000(C000,40)(C000,40)C000(C000,40)(C000,40)(C000,40)C000(C000,40)(C000,40)(C000,40))(C000(C000,10)(C000,40)(C000,40)C000(C000,10)(C000,40)(C000,40)C000(C000,10)(O0-10,10)(O000,20)C000(C000,40)(C000,40)C000(C000,40)(C000,40)C000(C000,40)(C000,40)C000(C000,40)(C000,40)(C000,40)C000(C000,40)(C000,40)(C000,40)))(1)(C000(C000(C000,40)(C000,40))(C000(C000,40)(C000,40)C000(C000,40)(C000,40)(C000,40))(C000(C000,40)(C000,40)C000(C000,40)(C000,40)C000(C000,40)(C000,40)C000(C000,40)(C000,40)C000(C000,40)(C000,40)(C000,40))(C000(C000,10)(C000,40)(C000,40)C000(C000,10)(C000,40)(C000,40)C000(C000,40)(C000,40)C000(C000,40)(C000,40)C000(C000,40)(C000,40)C000(C000,40)(C000,40)(C000,40)C000(C000,40)(C000,40)(C000,40))(C000(C000,10)(C000,10)C000(C000,40)(C000,40)C000(C000,40)(C000,40)C000(C000,40)(C000,40)C000(C000,40)(C000,40)C000(C000,40)(C000,40)(C000,40)C000(C000,40)(C000,40)(C000,40)))(1)(C000(C000(C000,40)(C000,40))(C000(C000,40)(C000,40)C000(C000,40)(C000,40)(C000,40))(C000(C000,40)(C000,40)C000(C000,40)(C000,40)C000(C000,40)(C000,40)C000(C000,40)(C000,40)C000(C000,40)(C000,40)(C000,40))(C000(C000,10)(C000,40)(C000,40)C000(C000,40)(C000,40)C000(C000,40)(C000,40)C000(C000,40)(C000,40)C000(C000,40)(C000,40)C000(C000,40)(C000,40)(C000,40)C000(C000,40)(C000,40)(C000,40))(C000(C000,10)(C000,10)C000(C000,40)(C000,40)C000(C000,40)(C000,40)C000(C000,40)(C000,40)C000(C000,40)(C000,40)C000(C000,40)(C000,40)(C000,40)C000(C000,40)(C000,40)(C000,40)))(1)(C000(C000(C000,40)(C000,40))(C000(C000,40)(C000,40)C000(C000,40)(C000,40))(C000(C000,10)(C000,40)(C000,40)C000(C000,40)(C000,40)C000(C000,40)(C000,40)C000(C000,40)(C000,40)(C000,40))(C000(C000,10)(C000,10)C000(C000,40)(C000,40)C000(C000,40)(C000,40)C000(C000,40)(C000,40)C000(C000,40)(C000,40)(C000,40)C000(C000,40)(C000,40)(C000,40))(C000(C000,10)(C000,40)(C000,40)C000(C000,10)(C000,40)(C000,40)C000(C000,10)(O0-10,10)(O000,20)C000(C000,40)(C000,40)C000(C000,40)(C000,40)C000(C000,40)(C000,40)(C000,40)C000(C000,40)(C000,40)(C000,40)))(1)(C000(C000(C000,40)(C000,40))(C000(C000,40)(C000,40)C000(C000,40)(C000,40))(C000(C000,40)(C000,40)C000(C000,40)(C000,40)C000(C000,40)(C000,40)C000(C000,40)(C000,40)(C000,40))(C000(C000,10)(C000,40)(C000,40)C000(C000,40)(C000,40)C000(C000,40)(C000,40)C000(C000,40)(C000,40)(C000,40)C000(C000,40)(C000,40)(C000,40))(C000(C000,10)(C000,10)C000(C000,40)(C000,40)C000(C000,40)(C000,40)C000(C000,40)(C000,40)C000(C000,40)(C000,40)(C000,40)C000(C000,40)(C000,40)(C000,40)))(1)(C000(C000(C000,40)(C000,40))(C000(C000,40)(C000,40)C000(C000,40)(C000,40))(C000(C000,40)(C000,40)C000(C000,40)(C000,40)C000(C000,40)(C000,40)C000(C000,40)(C000,40)(C000,40))(C000(C000,40)(C000,40)C000(C000,40)(C000,40)C000(C000,40)(C000,40)C000(C000,40)(C000,40)(C000,40)C000(C000,40)(C000,40)(C000,40))(C000(C000,10)(C000,40)(C000,40)C000(C000,40)(C000,40)C000(C000,40)(C000,40)C000(C000,40)(C000,40)(C000,40)C000(C000,40)(C000,40)(C000,40)))(1)(O0-10(O0-10(C000,10))(C000(C000,10)(O0-10,10)(O000,20))(C000(C000,10)(C000,10)O0-10(C000,10)O000(C000,20))(C000(C000,10)(C000,40)(C000,40)C000(C000,10)(O0-10,10)(O000,20)C000(C000,10)(O0-10,10)(O000,20))(C000(C000,10)(C000,10)C000(C000,40)(C000,40)C000(C000,40)(C000,40)(C000,40)))(1)(O000(O000(C000,20))(C000(C000,10)(O0-10,10)(O000,20))(C000(C000,10)(C000,10)O0-10(C000,10)O000(C000,20))(C000(C000,10)(C000,40)(C000,40)C000(C000,10)(O0-10,10)(O000,20)C000(C000,10)(O0-10,10)(O000,20))(C000(C000,10)(C000,10)C000(C000,40)(C000,40)C000(C000,40)(C000,40)(C000,40)))"

C.

**S Figure 1.** Derived coloring identifier. A) Loose coloring identifier for MetaCyc CPD-20570 and KEGG C13014; B) Tight coloring identifier for KEGG C13014; C) Tight coloring identifier for MetaCyc CPD-20570.

**
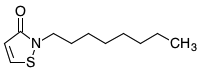
**

KEGG: C18752

**S Figure 2.** KEGG compound with S-containing aromatic ring.

**S Table 1.** Compounds with the same coloring identifiers, which includes R groups.

| **Databases** | **Tight coloring identifier** | **Loose coloring identifier** |
| --- | --- | --- |
| KEGG | 209 (1.1%) | 1132 (6.1%) |
| MetaCyc | 449 (2.4%) | 1638 (8.1%) |

KEGG: C02389

KEGG: C00530


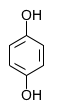

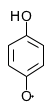

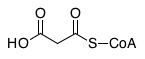

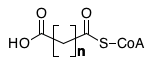


KEGG: C00083

KEGG: C03188


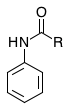

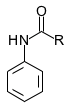


KEGG: C00484

KEGG: C01402


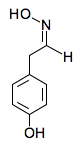

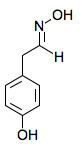


KEGG: C04353

KEGG: C04350


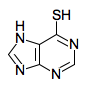

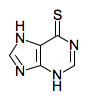


KEGG: C01756

KEGG: C02380

**A**

**B**

**D**

**C**

**E**

**S Figure 3.** Representative compounds that cannot be distinguished by coloring identifier. A) Compound and its radical form; B) Compound containing repeated substructure; C) Compound with R representing a generic group; D) Isomers containing C=N; E) Compounds after curation of aromatic substructures.


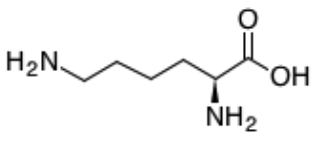


9

4

10

7

6

3

1

2

5

8

**S Figure 4.** KEGG Compound C00047.

**S Table 2.** Generation of atom identifiers for compound C00047 via graph coloring method.

| **Round** | **Atom identifier** | **Atom index** |
| --- | --- | --- |
| 1 | C | 1, 2, 3, 5, 8, 9 |
|  | N | 4, 10 |
|  | O | 6, 7 |
| 2 | C(C(C,1)(C,1)(N,1)) | 1 |
|  | C(C(C,1)(C,1)) | 2, 5, 8 |
|  | C(C(C,1)(O,1)(O,2)) | 3 |
|  | N(N(C,1)) | 4, 10 |
|  | O(O(C,1)) | 6 |
|  | O(O(C,2) | 7 |
|  | C(C(C,1)(N,1)) | 9 |
| 3 | C(C(C,1)(C,1)(N,1))(C(C,1)(C,1)C(C,1)(O,1)(O,2)N(C,1)) | 1 |
|  | C(C(C,1)(C,1))(C(C,1)(C,1)C(C,1)(C,1)(N,1)) | 2 |
|  | C(C(C,1)(O,1)(O,2))(C(C,1)(C,1)(N,1)O(C,1)O(C,2)) | 3 |
|  | N(N(C,1))(C(C,1)(C,1)(N,1)) | 4 |
|  | C(C(C,1)(C,1))(C(C,1)(C,1)C(C,1)(C,1)) | 5 |
|  | O(O(C,1))(C(C,1)(O,1)(O,2)) | 6 |
|  | O(O(C,2))(C(C,1)(O,1)(O,2)) | 7 |
|  | C(C(C,1)(C,1))(C(C,1)(C,1)C(C,1)(N,1)) | 8 |
|  | C(C(C,1)(N,1))(C(C,1)(C,1)N(C,1)) | 9 |
|  | N(N(C,1))(C(C,1)(N,1)) | 10 |
| Only chemical information of atom type and bond type is included in atom naming. The first three rounds of naming are shown above. | | |
